# Identifying patients with non-response for an extension of TMS treatments for depression: A retrospective analysis of clinical response conversion

**DOI:** 10.1016/j.transm.2025.100092

**Published:** 2025-04-18

**Authors:** Brandon J. Lew, Eric Tirrell, Andrew M. Fukuda, Nimalan Murugan, Carissa Perez, Wenricka Griffith, Linda L. Carpenter

**Affiliations:** a Butler Hospital TMS Clinic and Neuromodulation Research Facility, Providence, RI 02906, United States; b Brown Department of Psychiatry and Human Behavior, Providence, RI 02906, United States; c Kaiser Permanente, Fontana, CA 92553, United States

## Abstract

**Introduction:**

Transcranial Magnetic Stimulation (TMS) is an FDA-approved treatment for Major Depressive Disorder (MDD) which traditionally consists of 30 sessions of daily treatment followed by a taper phase of 6 sessions. Generally, over 50 % show a significant improvement in MDD symptoms; however, there are many patients who show a trend of improvement but have not have yet crossed that threshold defining meaningful clinical response. Extending the acute course with more sessions may optimize outcomes for the treatment course, however little data exist to inform which patients would benefit from treatment extension. In a retrospective analysis of naturalistic treatment data, we examined variables association with “conversion” of patients from nonresponder to responder status following extension of the acute course by at least 5 sessions.

**Methods:**

Response was defined as ≥ 50 % reduction from baseline score on the Inventory of Depressive Symptomatology-Self Report (IDS-SR). Of 485 treatment courses reviewed, we identified 40 patient series where a course extension (≥41 treatments) was given to patients who had not achieved response at treatment 30. In 21 treatment series, the patient “converted” to responder status by end of the course. Mixed model ANOVAs of IDSSR scores were used to compare the groups and identify features associated with conversion to response with additional sessions.

**Results:**

In 21 treatment series (representing 17 unique individuals), patients converted from nonresponse to response with additional treatment sessions beyond 30. Greater degree of IDS-SR improvement from baseline to treatment 30 was significantly associated with conversion to responder status. A 26 % reduction in IDS-SR total by treatment 30 showed 77.5 % accuracy in classifying whether or not a patient would ultimately respond to a 10-session course extension.

**Conclusion:**

Extension of the acute TMS course beyond 30 sessions is effective in converting a subset of nonresponding patients into treatment responders. The patients that were most likely to benefit from extension were those that improved the most by treatment 30. While these data may begin to inform extension criteria research, further study is needed to determine a clinically reliable threshold for selection of patients for an extension.

## Introduction

Major depressive disorder (MDD) is a common and debilitating disorder, with an estimated 12-month prevalence of medication-treated MDD in the United States of 8.9 million adults. Of these, an estimated 2.8 million (30.9 %) have treatment resistant depression, which is associated with major healthcare costs and unemployment (Zhdanava et al., 2021). Transcranial Magnetic Stimulation (TMS) is a safe and effective treatment for treatment resistant MDD, which may have a response rate of 58–83 % (Carpenter et al., 2012; Cole et al., 2020; Sackeim et al., 2020). Landmark studies in TMS treatment for MDD established that longer treatment courses showed greater response and remission rates, with the initial FDA approval from a prospective study showing greater response at 6 weeks compared to 4 weeks (O’Reardon et al., 2007). (Avery et al., 2008; O’Reardon et al., 2007). Given this, the most widely utilized TMS protocol involves 10 Hz stimulation to the left dorsolateral pre-frontal cortex for 30 treatments over 6 weeks, with an additional 6 taper phase treatments typically given over 3 weeks. In a large prospective trial, Avery et al. (2008) showed that extension of the acute treatment phase, i.e., continuing beyond treatment #30 with 5 TMS sessions/week for additional weeks up to session #60, was associated with increasing cumulative sustained response rates over time. A report by Hutton et al. (2023) also described a positive relationship in a large registry database between number of TMS sessions and magnitude of symptom reduction, with TMS treatments beyond #36 associated with additional benefit (Hutton et al., 2023).

In clinical practice, a decision must be made about when to stop the course of treatments for each individual patient. Many insurance policies require the course be limited to 30 acute phase (once daily) sessions followed by 6 taper treatments, but based on the “more is better” signals emerging from multiple studies, others permit extension of the acute course for additional weeks before the taper phase. A recent naturalistic study showed, that of 28 patients who were nonresponders following session #36, 54 % “converted” to response status with continuation of the course for up to 70 total sessions; however, the authors were unable to identify significant predictors of response to treatment extension (Razafsha et al., 2023). Whether treatment extension should be recommended for all MDD patients remains a pressing clinical question that the clinician must make following session 30 (end of the standard induction phase). We examined naturalistic TMS treatment data to better understand which patients may be good candidates for an extension.

## Methods

### Participants

Patients who received treatment at the Butler Hospital TMS Clinic for major depressive disorder (MDD) were included in this retrospective chart review analysis approved by the Butler Hospital Institutional Review Board. We extracted cases from treatment data captured between 2013 and 2023. All patients met insurance criteria for TMS coverage, i.e., primary diagnosis of MDD, resistance to two or more adequate antidepressant medication trials or a documented history of intolerance to antidepressant medications, absence of psychotic features, and a failed trial of psychotherapy. Diagnosis was made by clinical interview with a psychiatrist specializing in mood disorders, with collateral supporting information provided by the patient’s referring clinicians and medical treatment records. Patients eligible for treatment in this clinic met usual TMS safety criteria, e.g., absence of implanted intracranial ferromagnetic metal, absence of significant neurological disorders or seizure history. Per standard clinical practice, all patients were on stable medication regimens at time of TMS initiation and patients were directed not to change medications during the course of TMS therapy.

All patients received insurance pre-approval for 36 treatment sessions. A portion of patients had insurance coverage that permitted the treating physician to request an extension of the acute course with additional sessions beyond 36 when it was clinically warranted, i.e., there was some evidence of improvement (at least 25 % drop in depression scale scores from baseline, even if not sustained to session #30) but the patient had not yet achieved response or remission. Our clinic’s standard practice was to request insurance approval for up to 10 additional treatments; these were administered as 5/week until session #40, after which the 6-session taper phase took place over several weeks. For selection of cases for this analysis, an extended course of treatment was defined as a patient receiving ≥ 41 sessions.

Stimulation protocols varied, but typically consisted of 10 Hz rTMS to the left dorsolateral prefrontal cortex (DLPFC). Other protocols included iTBS to the left DLPFC, 5 Hz rTMS to the left DLPFC, and 1 Hz rTMS to the right DLPFC; all were delivered at intensity of 120 % relative to resting motor threshold. Changes in protocols were typically made due to poor tolerability of the initial 10 Hz protocol. Figure-8 coils were used to apply stimulation on Neurostar, Magventure, or Nexstim TMS device systems.

### Clinical Assessment

Depression severity was measured using the Inventory of Depressive Symptomatology-Self Report (IDS-SR) (Rush et al., 1996) scale at baseline (prior to first TMS), at treatment #30, and after the final session of the course (end of acute). Clinical response was defined as ≥ 50 % reduction in score from baseline to post-treatment. Conversion to responder status was defined as having a non-response at treatment 30 (< 50 % reduction at treatment 30) followed by reaching response (≥50 % reduction) after extension. Therefore, non-conversion was defined as having non-response at both treatment 30 and after extension. Remission on the IDS-SR was defined as final score ≤ 14. Patient Health Questionnaire-9 Item (PHQ-9) (Kroenke et al., 2001) scores were also collected at the same time points and analyzed in a post-hoc confirmatory analysis.

### Statistical analyses

Mixed model ANOVAs were used to compare the IDS-SR scores for two groups based on their outcome following treatment extension: nonresponders (non-converters) whose final IDS-SR score remained less than 50 % decreased from baseline versus responders (converters) whose symptom reduction did ultimately reach the 50 % IDS-SR criterion after TMS treatment extension using data from baseline and at treatment 30 (before extension). IDS-SR score was the dependent variable with repeated measure over time (baseline and treatment 30), and group was used as the independent predictor, with age as a covariate. Percent change from baseline at treatment 30 was also calculated and used to generate a receiver operating characteristic (ROC) curve. All data analysis was performed using R version 4.4.0 (R R Core Team, 2024). Mixed model ANOVAs were calculated with packages ‘rstatix’ (Kassambara, 2023) and visualized with ‘ggplot2’ (Wickham, 2016) and ‘ggstatsplot’ (Patil, 2021). ROC plots were created with R package ‘plotROC’ (Sachs, 2017). Post-hoc ANOVA models were run with PHQ-9 data from the same subjects.

## Results

### Treatment course selection

Of 54 courses identified as having a treatment extension (> 40 total treatment sessions in the course), 14 were in patients that had already met criteria for IDS-SR response by treatment 30 and their course was continued to optimize antidepressant effect with the goal of remission. Out of these 14 courses that showed response at treatment 30, n = 10 continued with improvement to remission status, n = 2 remained in response criteria without reaching remission, and n = 2 showed worsening to nonresponse. These courses were excluded for this analysis. The remaining 40 treatment courses, consisting of 34 unique patients, were selected for analysis (6 treatment courses were re-treatment in previously treated patients). Principal analyses included all 40 treatment courses, however follow up analyses were performed using only unique participants and excluded these re-treatment courses to ensure robustness of findings.

Of the 40 courses studied, all initiated the series with 10 Hz rTMS left DLPFC stimulation; 10 courses continued this protocol throughout the entirety of the course and another 18 had this protocol for a majority of sessions; 3 had a majority with iTBS, and 5 had a majority with 5 Hz stimulation. The remaining 4 had a majority of sessions with 1 Hz (right DLPFC) stimulation.

### Demographics and outcomes

Patients studied ranged in age from 19 to 72 years, with an average age of 41.8 years. A majority of patients identified as female (76 %). Patient demographics are noted in Table 1. For patients that had re-treatment courses, data from their initial treatment course was used for Table 1. Total number of treatments per course ranged from 41 to 59 sessions, with a median and mean of 46 treatments. Since we routinely requested insurance extensions for 10 additional sessions, the majority of courses (65 %) had 46 total sessions.

**Fig. 1.**
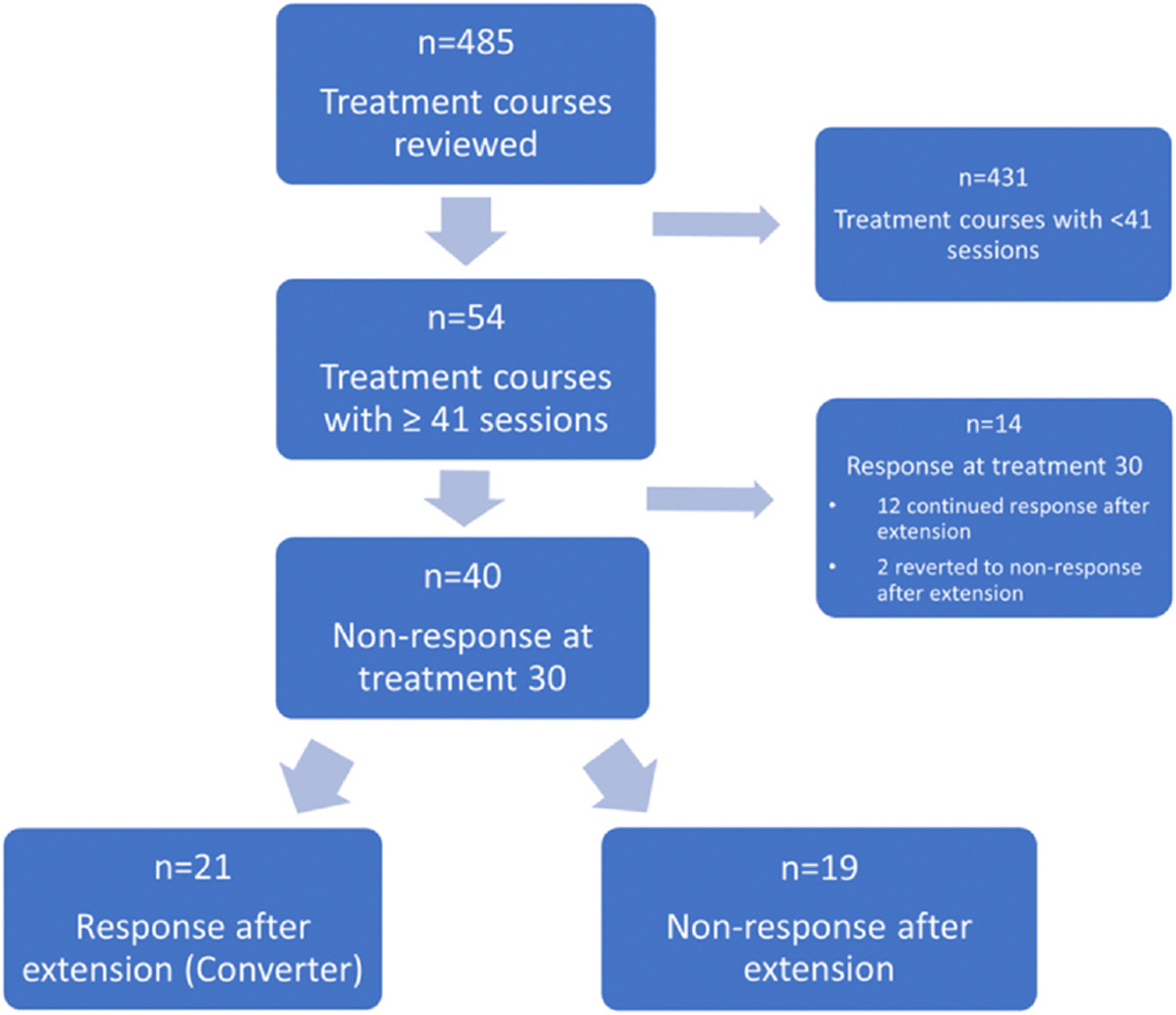
Summary of treatment course selection and respective groups.

Of 40 courses where the patient was a nonresponder immediately following session #30, 21 course extensions (52.5 %) resulted in conversion to response after extension, while 19 (47.5 %) did not (Fig. 1). Eight of these 40 extended courses (20 %) resulted in remission per IDS-SR criteria.

Comparison between patients that converted to responders versus those that did not showed a significant effect of age such that converters were more likely to be younger than non-converters (t(32) = −2.64, *p* = .0128). Converter and nonconverter groups did not show differences in sex, history of past psychiatric hospitalization, nor history of past ECT (*p* > .05). Significant differences were also not found between converters and nonconverters in total number of treatments/course nor in baseline MDD severity level (IDS-SR scores and PHQ-9 scores both *p* > .05; Table 1). Table 1 shows data comparing outcome groups with retreatment courses excluded from the sample. Subsequent comparisons include re-treatment courses.

To further investigate the effect of age, we ran a quantile regression with change in IDS-SR score from treatment 30 to post-extension as the dependent variable, and age as the independent variable. These results indicated no significant effect of age on change in IDS-SR score during the extension (t = 0.953, *p* = .345).

### IDS-SR scores over time in response outcome groups (converters vs. nonconverters) with acute course extension

Mixed model ANOVA of IDS-SR scores showed a significant group by time interaction (F(1,38) = 10.75, *p* = .003) revealing that patients who ended up converting from nonresponders to responders already had a relatively greater degree of improvement in IDS-SR from baseline at treatment 30, compared to patients who remained nonresponders following acute course extension. Models with PHQ-9 scores showed the same significant group-by-time interaction (F(1,30) = 8.94, *p* = 0.006) showing that a steeper trajectory of improvement on this scale from baseline to session 30 was also associated with ultimate response to treatment extension.

Four retreatment courses showed response again with a repeat extended course, however this was not the case for 2 retreatment courses. Analyses were repeated after removing multiple treatment courses per patient; the same interaction effects remained significant in models with both IDS-SR and PHQ-9 (IDS-SR: F(1,32) = 6.68, *p* = .015; PHQ-9: F(1,26) = 5.56, *p* = 0.026), reflecting the robustness of the association between steeper trajectory of improvement by treatment 30 and conversion to response after extension.

**Fig. 3.**
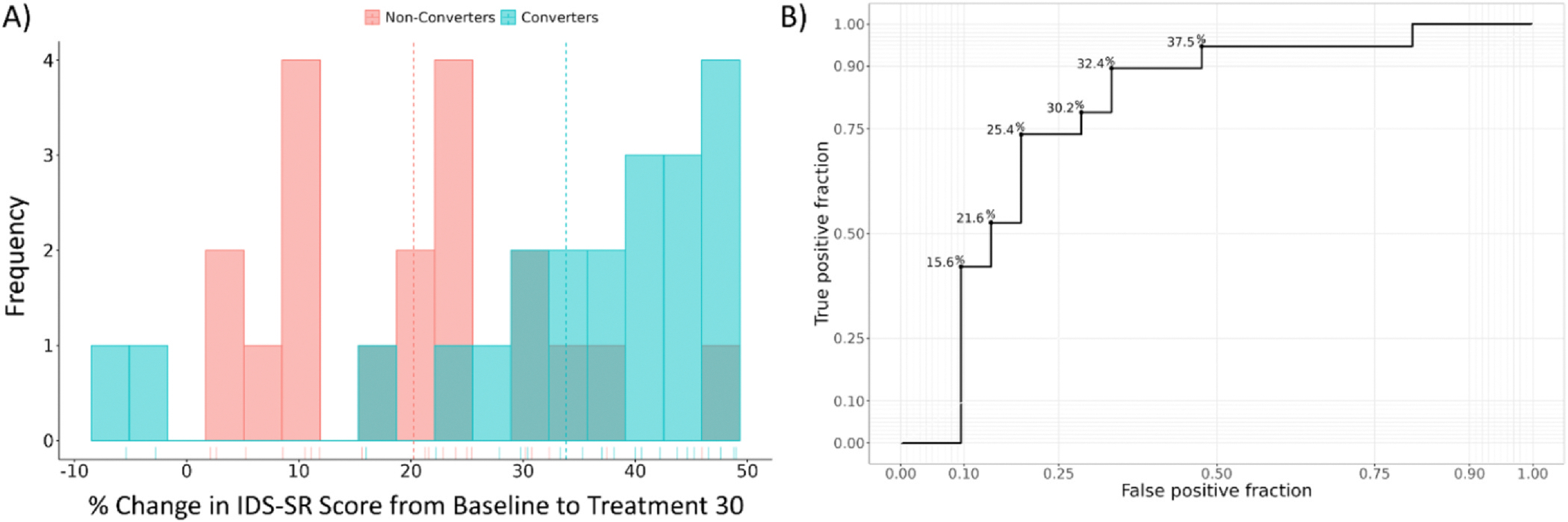
A) Frequency histogram of percent change in IDS-SR scores (from baseline) at treatment #30 for converters (n = 21, blue bars) versus non-converters (n = 19, red bars). Vertical dotted lines denote group means. B) Receiver Operating Characteristic (ROC) for percent change in IDS-SR score from baseline to treatment 30 predicting conversion to response. Accurate classification (with % improvement from baseline appearing in Fig. 3B at each horizontal step) indicates the percent change in IDS-SR score at session 30 that correctly classifies the patient as a converter following acute course extension, with true positive fraction (y-axis) showing the proportion of converters correctly identified using the data at treatment 30 and false positive fraction (x-axis) showing the proportion of non-converters incorrectly classified using the data at treatment 30.

On average, converters had a 33.8 % improvement in IDS-SR score from baseline at treatment 30, while non-converters had a 20.2 % improvement (Fig. 3A). For PHQ-9, converters had an average 44.7 % improvement from baseline compared to non-converters with a 22.8 % improvement from baseline at treatment 30. We conducted additional analyses to display the trade-off between a higher versus lower cutoff score in classifying converters versus non-converters using percent change. A receiver operating characteristic (ROC) curve was generated for percent change in IDS-SR from baseline to treatment 30 classifying conversion to response after extension, and the resulting curve had an area under the curve (AUC) of 0.787 (Fig. 3B). Cutoff scores of 26 % and 33 % improvement in IDS-SR score from baseline showed the highest degree of accuracy (77.5 %) in classifying cases that convert versus cases that do not. That is, a cutoff score of 26 % accurately classified 17/21 (81 %) converters and 14/19 (73 %) non-converters, while a cutoff score of 33 % accurately classified 14/21 (67 %) converters and 17/19 (89 %) non-converters.

We also ran an exploratory analysis examining data from earlier in the treatment course. Previous data has shown that early improvement in treatment course may be predictive of response outcomes (Feffer et al., 2018). A subset of participants in this dataset had available scores from session 20, and repeat analysis using session 20 IDS-SR scores instead of session 30 scores showed no significant group by time interaction (F (1,29) = 1.587, p = .218).

Finally, we ran an additional exploratory analyses reintroducing cases that had previously shown response, We examined the association between pre-extension improvement and change in scores by the end of the extension. We performed a linear regression with change in IDS-SR score from baseline to treatment 30 as the dependent variable and change in IDS-SR score from treatment 30 to post-extension as the independent variable, using all courses that received an extension (regardless of status at treatment 30). This model showed a significant relationship suggesting that greater improvement in the standard course is associated with greater improvement following extension (t (52) = 2.04, p = .047).

## Discussion

In the current study, we examined naturalistic treatment outcomes for courses of TMS treatment for MDD to determine which, if any, factors were associated with response to an extension of a standard treatment course. Approximately half of treatment extensions resulted in conversion to treatment response. Patients whose symptom scores converted to response status after an extension were more likely to have had a greater degree of improvement in symptoms from baseline to treatment 30 and to be younger in age. Conversely, factors which did not significantly correspond to treatment response were baseline scores on IDS-SR or PHQ-9. This suggests that severity of depression at pre-treatment baseline does not have a large impact on whether an extension is likely to be helpful. Instead, it is the amount of change from that individual’s baseline score over the first 6 weeks which matters more; presumably these data reflect the degree of symptom improvement that has occurred by session 30. Our finding that younger age is a positive predictor of response conversion does not readily translate to clinical application, since there was a considerable age range in both groups and there was no significant association between age and improvement in scores during the extension.

How might these results be used to move towards understanding a threshold at session #30 that supports recommendation of an acute course extension? We found that either 26 % or 33 % decrease in IDS-SR total by treatment 30 showed the highest accuracy (77.5 % accurate) in classifying whether or not a patient would ultimately respond to a 10-session course extension. Our ROC curve illustrated this trade-off, where a lower threshold more accurately includes patients that convert, while a higher cutoff more accurately excludes patients that do not convert to response. We note that our study is limited in sample size, and no cutoff was 100 % accurate in predicting conversion to response; two patients that ultimately benefitted from a treatment extension had relatively limited improvement in symptoms by treatment 30, and one patient that had a relatively good level of improvement by treatment 30 ultimately failed to achieve 50 % response following the extension. A 10-session extension may still benefit some patients who have shown minimal to modest improvement by #30, so the clinician must incorporate numerous considerations and apply a personalized risk-benefit ratio when deciding whether extension of the acute course is a reasonable approach.

The improvement in response outcomes that we observed with course extensions aligns with numerous reports showing enhanced antidepressant effects related to cumulative TMS exposure. Several studies suggest that increasing number of pulses per session is associated with better outcomes (Fitzgerald et al., 2020; Sackeim et al., 2020) when delivering once-daily sessions. Accelerated protocols increase both the number of sessions and pulses per day, often leading to faster and improved outcomes (Caulfield et al., 2022; Cole et al., 2022, 2020). A convergence of varied approaches to TMS suggests that desirable treatment outcomes may be achieved through more cumulative stimulation exposure (Van Rooij et al., 2024). A recent naturalistic study of over 7000 patients found steady, continued improvement (PHQ-9 scores) over time after session 36, without evidence of plateau; in a subset of 629 patients who received a total of 37–41 sessions, the response and remission rates increased from 46 % and 15 %, respectively, (at session 30) to 62 % and 30 % after the final session (Hutton et al., 2023). Higher endpoint response and remissions rates were not seen in a different subgroup (n = 349) that received more than 41 sessions in their acute course, reflecting a likely pattern diminishing returns at some point. Interestingly, our conversion response rate of 53 % to extension closely resembles another recent naturalistic treatment study which showed 54 % conversion response with extension beyond 36 sessions (Razafsha et al., 2023). This alignment validates the notion that it is worthwhile to recommend more than 36 TMS sessions for some depressed patients. While the Razafsha analysis could not identify significant predictors of response to extension, they did not specifically examine the degree of improvement in depressive symptoms following session 30.

While our findings contribute to the growing body of work showing an association between higher response rates and greater cumulative stimulation exposure, we note that all naturalistic treatment data reflect inherent biases, as a clinician’s decision to extend a patient’s acute course is more likely to happen when there is evidence of at least some degree of clinical progress by the end of week 6. Nevertheless, previously published studies of course extension have not identified predictors of conversion from nonresponders to responders, and our findings may be considered clinically actionable, providing guidance for a decision/recommendation that must be made for nearly every MDD patient during acute TMS care. A data-informed recommendation for extension may be considered more prudent than treatment of all TMS patients with additional sessions, as the latter could result in inefficient use of time, money, and other resources for those who will ultimately not achieve substantive MDD symptom relief.

It is important to note the limitations of our study. Given the retrospective naturalistic analysis, we are unable to prospectively control or manipulate eligibility criteria for a treatment extension. While some patients had early response and terminated their courses prior to session 30, patients selected for extension typically had some benefit from the first 6 weeks of once-daily stimulation. Furthermore, insurance companies for about one-third of our clinic sample (including federal healthcare policies) will not consider requests for extension of the acute course with more sessions beyond 36. These circumstances may have introduced biases that led to inflation of treatment outcomes. Naturalistic treatment in clinical settings does not reflect uniform application of the same stimulation protocol for all patients, and we did not control for the variability in treatment protocols in our analyses. Our findings predicting treatment extension outcomes may not be generalizable to any one specific protocol or to accelerated protocols. It should also be mentioned that change in self-report scale scores do not always capture the level of disability or functional improvement that patients experience over time. For some patients, a partial response characterized by 30 % drop form baseline score may correspond with clinically meaningful benefit. Our sample size was also relatively limited, with several retreatment courses included in the initial analysis. Importantly however, our findings held when excluding retreatment courses. Finally, concurrent psychotropic medication use may have played a role in response outcomes, and physical and psychiatric comorobidities were not examined in our analyses. Future study is needed with larger sample sizes to both replicate our findings, and understand how this relationship may be impacted by different comorbidities or medication regimens.

In summary, we found TMS treatment extension can continue to build upon antidepressant effects, with about half of patients achieving response at treatment endpoint despite not having achieved it at session 30 – the point when they would typically start the final taper phase with 6 final treatments over several weeks. Controlled prospective studies that randomize patients to treatment courses with differing numbers of total sessions would be ideal to confirm these findings. Development of biomarkers that signal maximal antidepressant benefit or the need to change stimulation target would also be helpful, as occasionally the continuation of daily sessions is associated with a trajectory of early response that is not sustained over subsequent weeks and a plateau or subsequent symptom worsening is seen. Given the increasing use of TMS in clinical settings worldwide, large, prospective studies will be needed to identify reliable predictors of response for TMS and other interventions, with specific focus on essential measurements and decision-points in the course of routine patient care.

## Figures and Tables

**Fig. 2.**
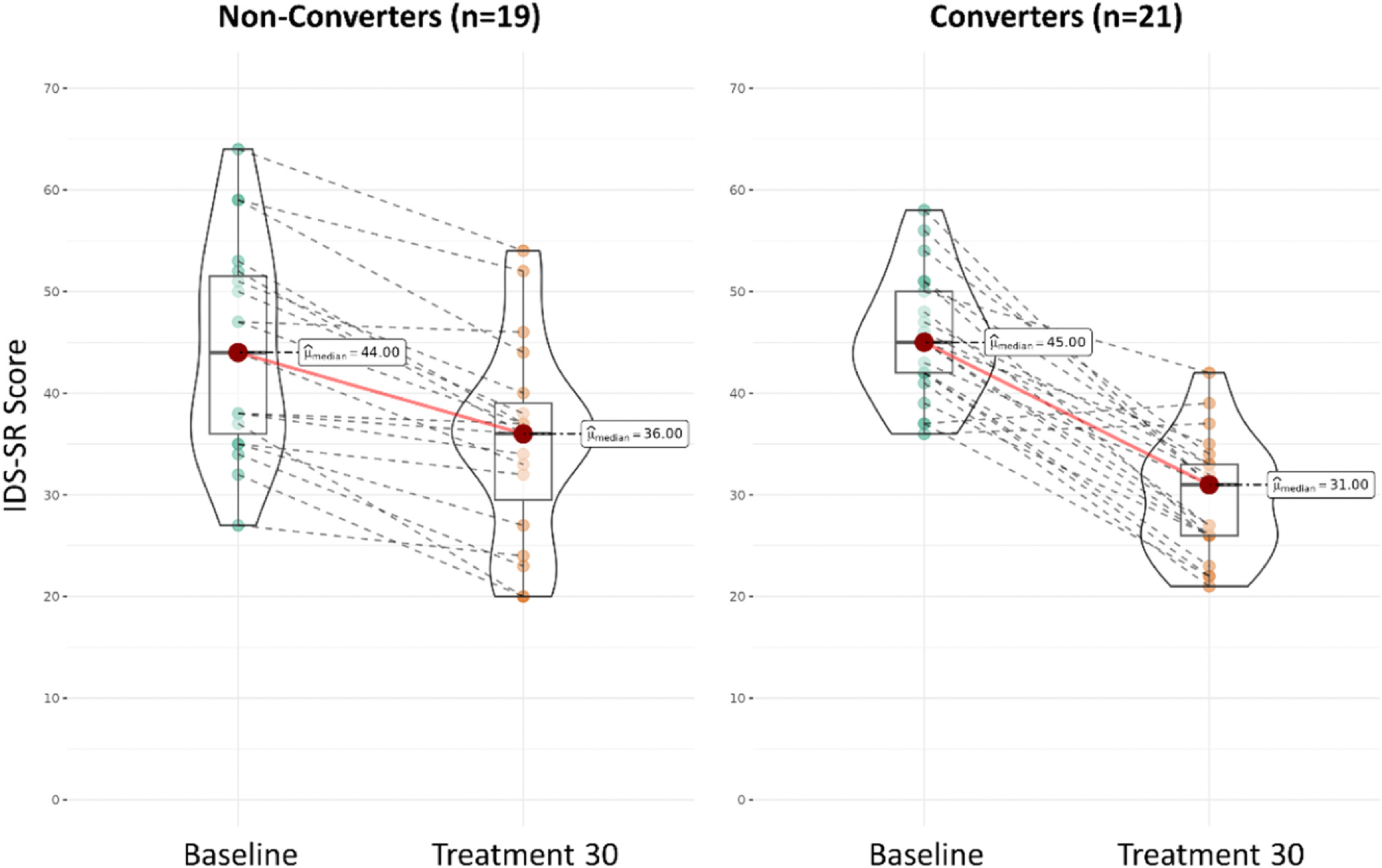
IDS-SR Scores at baseline and treatment 30 for courses ending in treatment response (Converters; n = 21, right) versus those that continued with nonresponse (Non-converters; n = 19, left). Converters had greater improvement in IDS-SR score by treatment 30 compared to non-converters.

**Table 1:** Clinical and Treatment Characteristics.

	Non-Converters (n = 17)	Converters (n = 17)	Sig (p)

Sex	3 M / 14 F	5 M / 12 F	.686
**Age [mean (SD)]**	**49.59 (12.1)**	**37.88 (13.8)**	**.013** *
History of Past Psychiatric Hospitalization(s) (n,%)	9 (52.9 %)	8 (47.1 %)	.999
History of Past ECT*(n,%)	3 (17.6 %)	0 (0 %)	.226
Total number of treatments (mean [SD])	45.18 (3.73)	46.04 (1.82)	.223
Baseline IDS-SR (mean [SD])	45.65 (9.89)	43.53 (4.76)	.432
Final IDS-SR (mean [SD])	33.06 (8.23)	16.47 (4.73)	< .001
Baseline PHQ-9 (mean [SD])	17.59 (4.78)	17.59 (2.55)	1.0
Final PHQ-9 (mean [SD])	10.65 (5.43)	4.82 (1.85)	< .001

* Past ECT exposure may have occurred in the current episode or in a prior depressive episode; this does not indicate completion of an adequate ECT trial nor failure to respond to ECT.

## References

[R1] Avery DH, Isenberg KE, Sampson SM, Janicak PG, Lisanby SH, Maixner DF, Loo C, Thase ME, Demitrack MA, & George MS (2008). Transcranial magnetic stimulation in the acute treatment of major depressive disorder: clinical response in an open-label extension trial. J Clin Psychiatry, 69, 441–451. 10.4088/JCP.v69n0315 18294022

[R2] Carpenter LL, Janicak PG, Aaronson ST, Boyadjis T, Brock DG, Cook IA, Dunner DL, Lanocha K, Solvason HB, & Demitrack MA (2012). Transcranial magnetic stimulation (TMS) for major depression: a multisite, naturalistic, observational study of acute treatment outcomes in clinical practice: research article: an observational study of TMS. Depress Anxiety, 29, 587–596. 10.1002/da.21969 22689344

[R3] Caulfield KA, Fleischmann HH, George MS, & McTeague LM (2022). A transdiagnostic review of safety, efficacy, and parameter space in accelerated transcranial magnetic stimulation. Journal of Psychiatric Research, 152, 384–396. 10.1016/j.jpsychires.2022.06.038 35816982 PMC10029148

[R4] Cole EJ, Phillips AL, Bentzley BS, Stimpson KH, Nejad R, Barmak F, Veerapal C, Khan N, Cherian K, Felber E, Brown R, Choi E, King S, Pankow H, Bishop JH, Azeez A, Coetzee J, Rapier R, Odenwald N, Carreon D, Hawkins J, Chang M, Keller J, Raj K, DeBattista C, Jo B, Espil FM, Schatzberg AF, Sudheimer KD, & Williams NR (2022). Stanford Neuromodulation Therapy (SNT): a double-blind randomized controlled trial. AJP, 179, 132–141. 10.1176/appi.ajp.2021.20101429 34711062

[R5] Cole EJ, Stimpson KH, Bentzley BS, Gulser M, Cherian K, Tischler C, Nejad R, Pankow H, Choi E, Aaron H, Espil FM, Pannu J, Xiao X, Duvio D, Solvason HB, Hawkins J, Guerra A, Jo B, Raj KS, Phillips AL, Barmak F, Bishop JH, Coetzee JP, DeBattista C, Keller J, Schatzberg AF, Sudheimer KD, & Williams NR (2020). Stanford accelerated intelligent neuromodulation therapy for treatment-resistant depression. AJP, 177, 716–726. 10.1176/appi.ajp.2019.19070720 32252538

[R6] Feffer K, Lee HH, Mansouri F, Giacobbe P, Vila-Rodriguez F, Kennedy SH, Daskalakis ZJ, Blumberger DM, & Downar J (2018). Early symptom improvement at 10 sessions as a predictor of rTMS treatment outcome in major depression. Brain Stimulation, 11, 181–189. 10.1016/j.brs.2017.10.010 29107623

[R7] Fitzgerald PB, Hoy KE, Reynolds J, Singh A, Gunewardene R, Slack C, Ibrahim S, & Daskalakis ZJ (2020). A pragmatic randomized controlled trial exploring the relationship between pulse number and response to repetitive transcranial magnetic stimulation treatment in depression. Brain Stimulation, 13, 145–152. 10.1016/j.brs.2019.09.001 31521543

[R8] Hutton TM, Aaronson ST, Carpenter LL, Pages K, Krantz D, Lucas L, Chen B, & Sackeim HA (2023). Dosing transcranial magnetic stimulation in major depressive disorder: Relations between number of treatment sessions and effectiveness in a large patient registry. Brain Stimulation, 16, 1510–1521. 10.1016/j.brs.2023.10.001 37827360

[R9] Kassambara A (2023). rstatix: Pipe-Friendly. Framework for Basic Statistical Tests.

[R10] Kroenke K, Spitzer RL, & Williams JBW (2001). The PHQ-9: Validity of a brief depression severity measure. J Gen Intern Med, 16, 606–613. 10.1046/j.1525-1497.2001.016009606.x 11556941 PMC1495268

[R11] O’Reardon JP, Solvason HB, Janicak PG, Sampson S, Isenberg KE, Nahas Z, McDonald WM, Avery D, Fitzgerald PB, Loo C, Demitrack MA, George MS, & Sackeim HA (2007). Efficacy and safety of transcranial magnetic stimulation in the acute treatment of major depression: a multisite randomized controlled trial. Biological Psychiatry, 62, 1208–1216. 10.1016/j.biopsych.2007.01.018 17573044

[R12] Patil I (2021). Visualizations with statistical details: The “ggstatsplot” approach. Journal of Open Source Software, 6, 3167. 10.21105/joss.03167

[R13] R Core Team (2024). R: A Language and Environment for Statistical Computing. Vienna, Austria: R Foundation for Statistical Computing.

[R14] Razafsha M, Barbour T, Uribe S, Behforuzi H, & Camprodon JA (2023). Extension of transcranial magnetic stimulation treatment for depression in non-responders: results of a naturalistic study. Journal of Psychiatric Research, 158, 314–318. 10.1016/j.jpsychires.2022.12.038 36628873

[R15] Rush AJ, Gullion CM, Basco MR, Jarrett RB, & Trivedi MH (1996). The inventory of depressive symptomatology (IDS): psychometric properties. Psychol Med. 26, 477–486. 10.1017/S0033291700035558 8733206

[R16] Sachs MC (2017). plotROC: a tool for plotting ROC Curves. Journal of Statistical Software, 79, 1–19. 10.18637/jss.v079.c02 30686944 PMC6347406

[R17] Sackeim HA, Aaronson ST, Carpenter LL, Hutton TM, Mina M, Pages K, Verdoliva S, & West WS (2020). Clinical outcomes in a large registry of patients with major depressive disorder treated with transcranial magnetic stimulation. Journal of Affective Disorders, 277, 65–74. 10.1016/j.jad.2020.08.005 32799106

[R18] Van Rooij SJH, Arulpragasam AR, McDonald WM, & Philip NS (2024). Accelerated TMS - moving quickly into the future of depression treatment. Neuropsychopharmacol, 49, 128–137. 10.1038/s41386-023-01599-z PMC10700378 37217771

[R19] Wickham H (2016). ggplot2: Elegant Graphics for Data Analysis. New York: Springer-Verlag.

[R20] Zhdanava M, Pilon D, Ghelerter I, Chow W, Joshi K, Lefebvre P, & Sheehan JJ (2021). The prevalence and national burden of treatment-resistant depression and major depressive disorder in the United States. J Clin Psychiatry, 82. 10.4088/JCP.20m13699 33989464

